# Hyperammonemia: An Unusual Cause of Encephalopathy in Multiple Myeloma

**DOI:** 10.7759/cureus.90707

**Published:** 2025-08-21

**Authors:** Aneal S Dayal, Moni Roy, Tulika Chatterjee

**Affiliations:** 1 Internal Medicine, University of Illinois College of Medicine Peoria, Peoria, USA

**Keywords:** african american, atypical back pain, chemotherapy, chemotherapy response, hyperammonemia, hyperammonemic encephalopathy, multiple myeloma

## Abstract

Multiple myeloma (MM) is a malignancy of plasma cells that commonly presents with kidney dysfunction, hypercalcemia, bone pain, and anemia. Although many patients exhibit these symptoms, other, less obvious presentations can complicate the prognosis. In this case report, we describe a 65-year-old African American female with known IgG Kappa MM, who initially presented with back pain - a common symptom associated with bone involvement in MM. However, during her hospital stay, she developed a profoundly altered mental status, which was later linked to elevated ammonia levels. Hyperammonemia is a rare but highly fatal complication in MM. Thus, all providers must be able to clinically recognize and understand how to potentially treat hyperammonemic encephalopathy in MM early in its course.

## Introduction

Multiple myeloma (MM) is a malignancy of plasma cells that most frequently presents as impairment of the kidneys, hypercalcemia, anemia, and bone pain [1]. Complications of MM vary but include bleeding/coagulopathy, osteolytic bone disease, spinal cord compression, renal injury, adrenal insufficiency, and venous thromboembolism [2]. However, MM can also manifest in ways that are not as typical, making the recognition and subsequent management of this disease challenging. Some of these uncommon presentations include pancreatitis, mesenteric ischemia, orbital disease, and vertigo [3].

One of the rarest presentations and complications of MM is hyperammonemia. The clinical presentation of hyperammonemia may vary but often includes altered mental status. Identifiable symptoms also include gait abnormalities, headache, nausea, and vomiting, with more severe levels presenting with encephalopathy, unresponsiveness, and seizures [4]. With a mortality rate of around 48% in those with MM, it is imperative to recognize hyperammonemia and act swiftly [5]. Among those diagnosed, the African American population and those in their 70s tend to be the most predominantly affected [5].

In this case report, we present a case of an African American female with known MM who developed acute encephalopathy due to MM-associated hyperammonemia during her admission. Despite what appeared to be a stable presentation, her condition rapidly deteriorated, highlighting the aggressive nature of this rare complication. This case underscores the importance of early recognition and intervention in patients with MM who develop unique and unexplained symptoms. It also highlights the need for further studies and literature to better understand the etiology and management options for this condition.

## Case presentation

A 65-year-old African American woman with a medical history of type 2 diabetes mellitus, osteoarthritis, hypertension, and fibromyalgia was diagnosed with IgG kappa MM two years prior to her current presentation. She initially responded well to first-line treatment with daratumumab, bortezomib, lenalidomide, and dexamethasone for approximately 20 months. This regimen was continued until disease relapse at that time, which occurred after noted symptoms of worsening diffuse pain, weakness, and subjective fevers. In response to relapse, her treatment was switched to a regimen of carfilzomib, pomalidomide, and dexamethasone.

**Figure 1:**
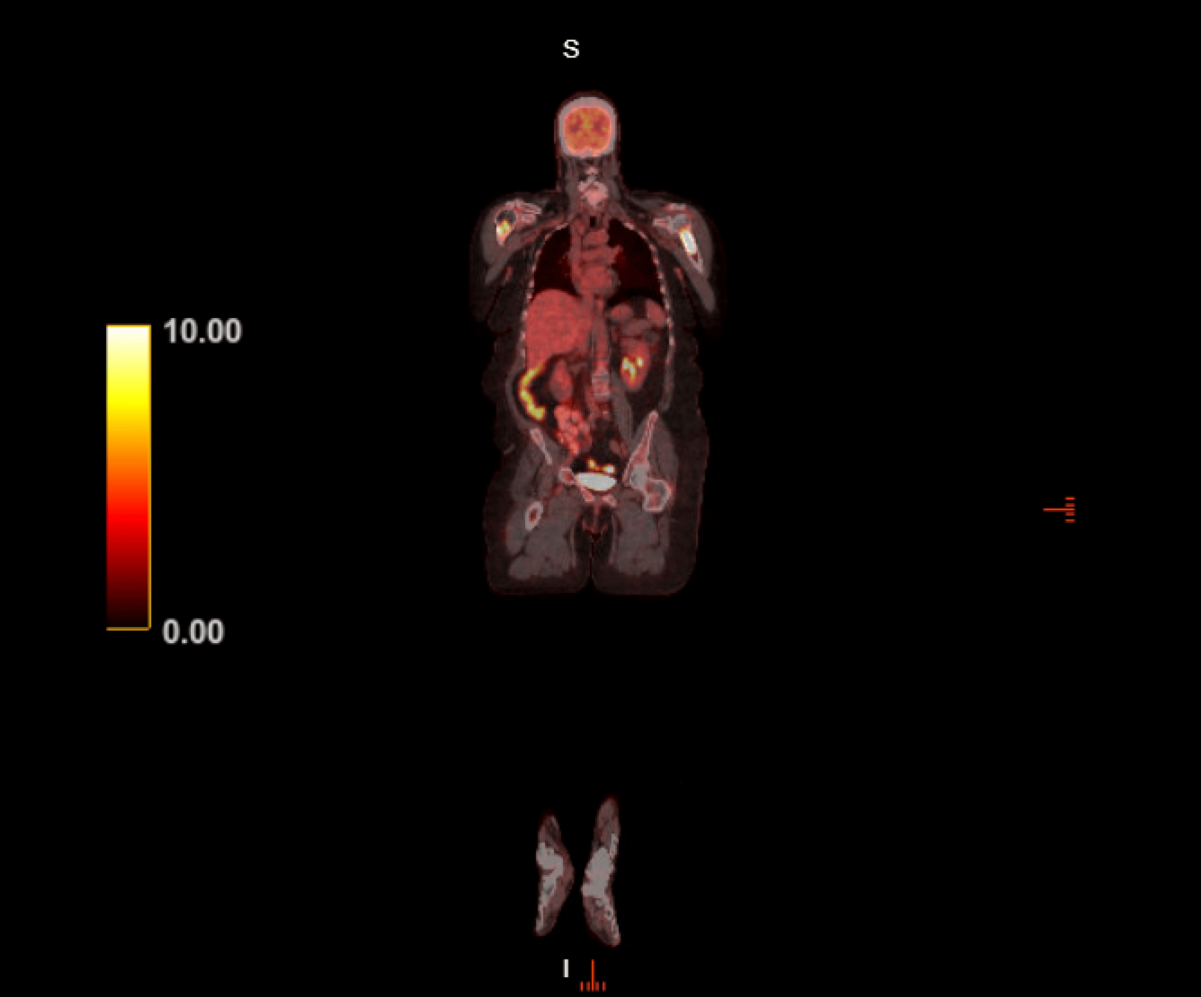
Positron emission tomography CT scan three months prior to admission with scattered lesions demonstrating FDG uptake FDG: fluorodeoxyglucose, CT: computed tomography

**Figure 2:**
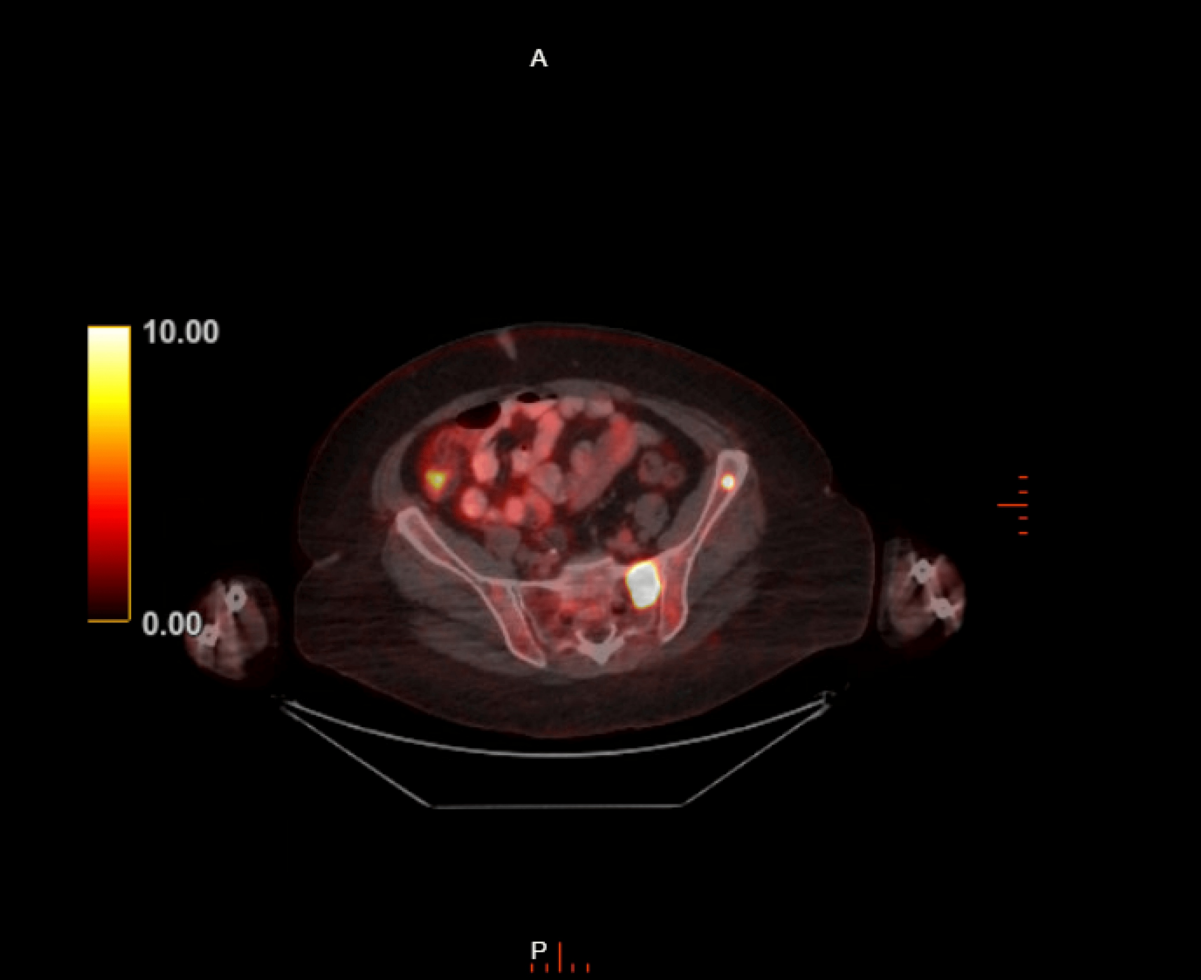
Positron emission tomography CT scan with focally FDG-avid lesion within the left iliac wing FDG: fluorodeoxyglucose, CT: computed tomography

**Figure 3:**
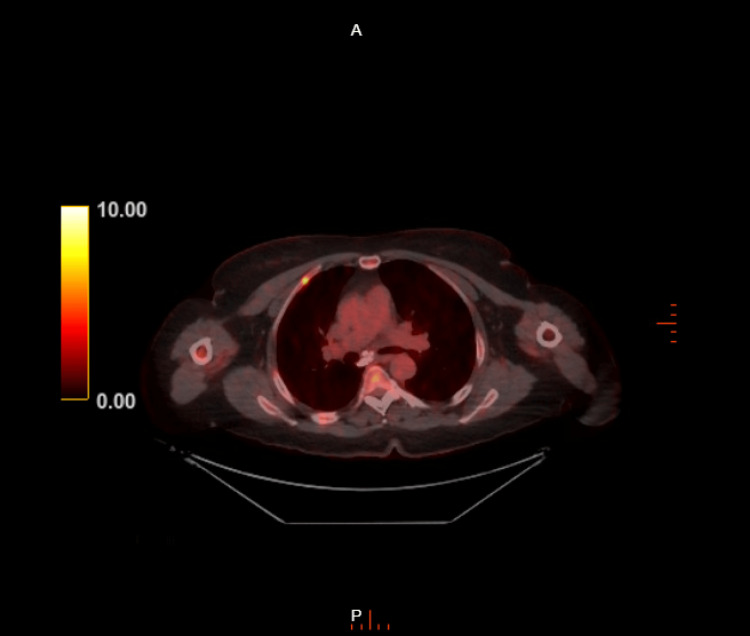
Positron emission tomography CT scan with FDG-avid lesion in the T5 vertebral body FDG: fluorodeoxyglucose, CT: computed tomography

Three months prior to presentation, a positron emission tomography (PET) CT scan revealed the progression of FDG-avid MM with increased intensity of FDG uptake in several pre-existing bone lesions and the development of new scattered bone lesions (Figure 1). Notably, she had confirmed lytic lesions in the left iliac wing (Figure 2) and thoracolumbar spine, particularly at T5-T7 (Figure 3).

One month prior to admission, the patient lifted heavy boxes, which led to a gradual increase in lower back pain. Despite completing her second cycle of carfilzomib and dexamethasone three days before presentation and ongoing plans to continue Pomalidomide for 21 days, her pain worsened. She subsequently presented to the emergency department with progressively worsening back pain. At the time of admission, her chronic medications included lisinopril, amlodipine, glipizide, and metformin.

Upon admission, the patient was hemodynamically stable with normal vital signs. Physical examination revealed bony tenderness to palpation over the lumbar spine and left hip, pain with left leg straight raise beyond 45 degrees, and intact sensation in all extremities. Laboratory tests showed anemia, thrombocytopenia, and leukopenia, which were all consistent with the patient's ongoing chemotherapy and previous laboratory results. The comprehensive metabolic panel was notable for an elevated gamma gap of 7 mg/dl, consistent with her known MM diagnosis (Table 1). A lumbar spine X-ray additionally revealed no acute fractures or subluxations. She was treated with non-opioid pain medications, and her back pain improved over the next few days.

**Table 1:** Pertinent laboratory values on admission vs. the start of encephalopathy

Laboratory	Normal range	Values on admission	Values when encephalopathy started
Hemoglobin	12.0-15.8 g/dL	8.2 g/dL	7.7 g/dL
Platelets	140-440 10(3)/mcL	53 10(3)/mcL	87 10(3)/mcL
White blood cells	4.00-12.00 10(3)/mcL	2.43 10(3)/mcL	5.17 10(3)/mcL
Neutrophils	47.0-73.0%	20.60%	25.30%
Lymphocytes	18.0-42.0%	71.20%	51.10%
Sodium	136-145 mmol/L	135 mmol/L	141 mmol/L
Potassium	3.5-5.1 mmol/L	3.9 mmol/L	3.3 mmol/L
Chloride	98-107 mmol/L	111 mmol/L	121 mmol/L
Bicarbonate	22-30 mmol/L	20 mmol/L	19 mmol/L
Total protein	6.3-8.2 g/dL	9.6 g/dL	10.6 g/dL
Albumin	3.5-5.0 g/dL	2.6 g/dL	2.7 g/dL
Glucose	70-99 mg/dL	251 mg/dL	207 mg/dL
Calcium	8.7-10.5 mg/dL	8.5 mg/dL	10.6 mg/dL
Ammonia	18-72 umol/L	NA	121 umol/L
Creatinine	0.6-1.1 mg/dL	0.88 mg/dL	1.07 mg/dL
Blood urea nitrogen	7-18 mg/dL	6 mg/dL	7 mg/dL
Lactic acid	0.5-2.2 mmol/L	0.5 mmol/L	NA
Aspartate aminotransferase	10-40 U/L	32 U/L	34 U/L
Alanine aminotransferase	7-56 U/L	17 U/L	21 U/L

**Figure 4:**
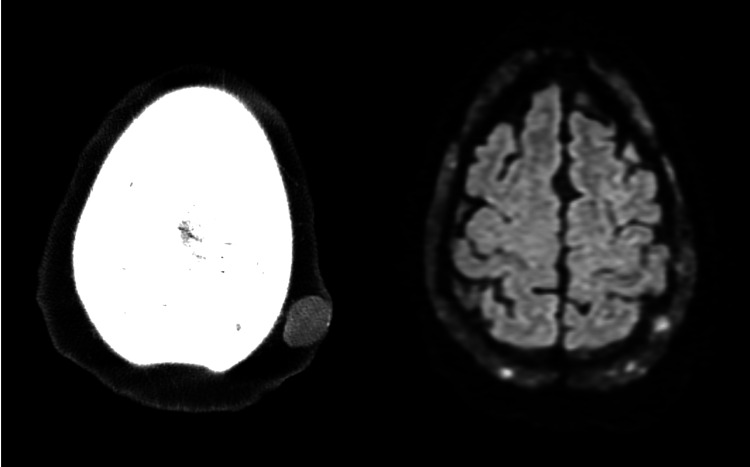
CT of the head revealing left parietal nonenhancing solid nodule (left image) and lytic lesions (right image) CT: computed tomography

**Figure 5:**
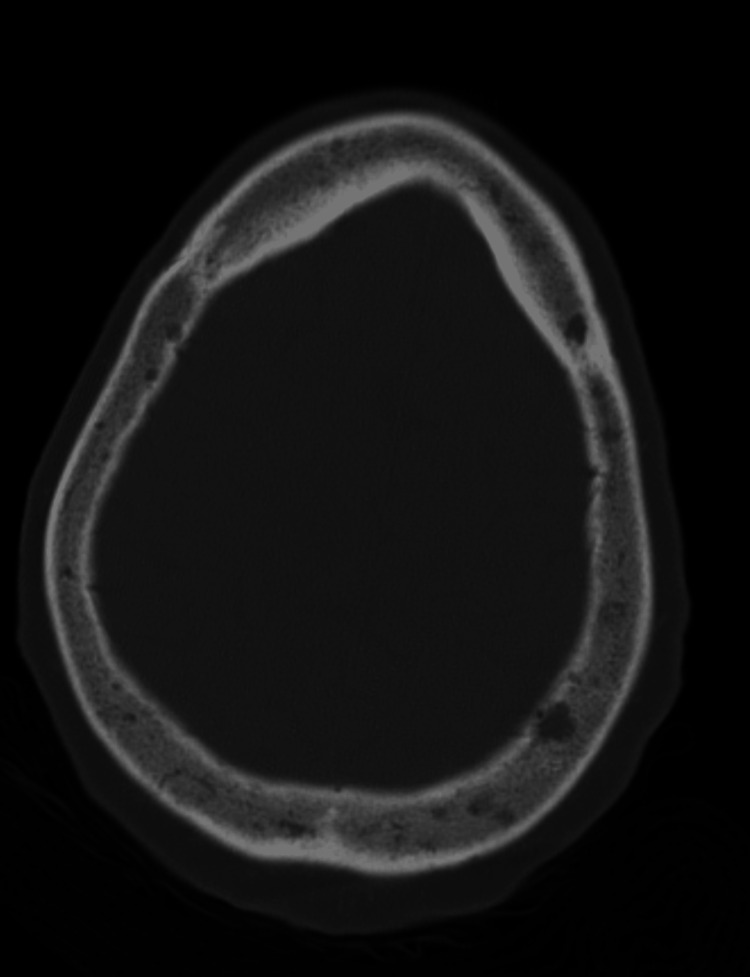
CT of the head demonstrating lytic lesions CT: computed tomography

**Figure 6:**
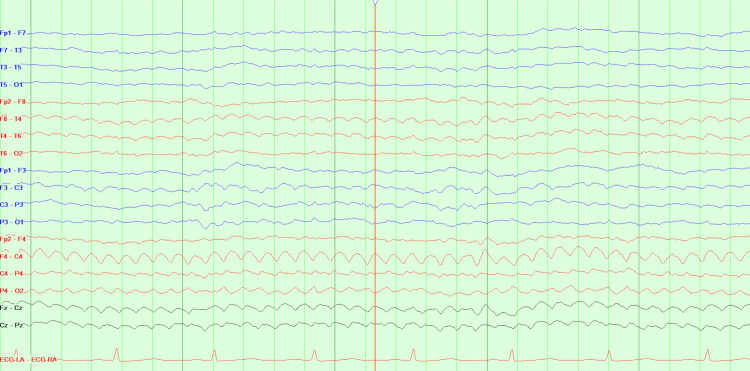
EEG demonstrating triphasic waves and diffuse background slowing EEG: electroencephalogram

However, on day five of her hospital stay, while awaiting placement in a skilled nursing facility, the patient developed new, sudden-onset neurological symptoms, including left facial droop, right gaze preference, generalized weakness, lethargy, mild aphasia, and dysarthria. Computed tomography (CT) of the head (Figures 4, 5) showed no acute intracranial abnormalities but did reveal lytic lesions and a solid nodule that was 18-fluorodeoxyglucose-avid, which was present on the prior positron emission tomography (PET) scans. A CT angiography of the head and neck was unremarkable, and a magnetic resonance imaging (MRI) of the brain showed no acute changes/findings and was only consistent with the patient’s history of MM. Those MRI findings show no acute intracranial hemorrhage, infarct, or abnormal enhancement, and diffuse bone marrow signal abnormality with superimposed, more focally prominent enhancing lesions compatible with a fatty marrow replacement or infiltrative process such as MM. As a result of the sudden onset of neurological symptoms, an electroencephalogram (EEG) was ordered. The EEG (Figure 6) was significant for triphasic waves and diffuse background slowing with poor regionalization, indicative of moderate encephalopathy, due to diffuse cerebral dysfunction likely secondary to an underlying metabolic etiology. There were no epileptiform features present to suggest an underlying epileptic disturbance.

**Figure 7:**
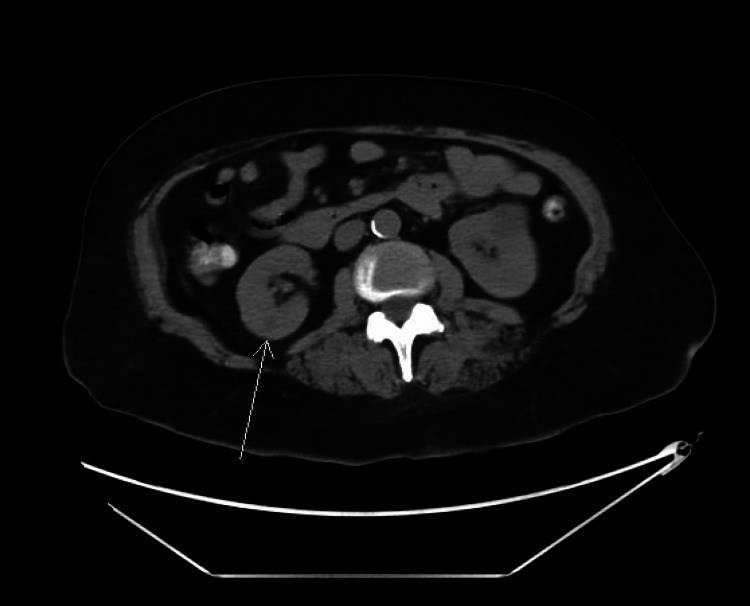
CT of the abdomen and pelvis demonstrating indeterminate small structure in the kidney CT: computed tomography

On repeat examination, the patient’s condition had significantly worsened, with diminished mental status and inability to follow commands or respond appropriately to questions. She demonstrated an inability to lift extremities when prompted, but did withdraw to painful stimuli in all extremities. Repeat labs revealed a lack of hypoglycemia, no electrolyte abnormalities, no fever, nor leukocytosis, subsequently ruling out various potential causes of her altered mental status (Table 1). Urinalysis and chest X-ray were without any signs of infection, aspiration, or any other acute findings. Blood cultures demonstrated no growth as well. The patient’s creatinine function worsened to 1.64 mg/dL from 1.39 mg/dL the prior day in the context of a baseline creatinine of 0.8 mg/dL. A CT of the abdomen and pelvis was performed in the setting of abdominal tenderness, which revealed indeterminate small structures in the kidney, including a 2.5-centimeter partially exophytic cyst in the interpolar region of the left kidney and a poorly visualized hypodense structure in the interpolar region of the right kidney (Figure 7). Elevated free kappa light chain levels were noted at 1097.76 mg/L, with decreased free lambda light chains. A paraneoplastic panel was obtained, which was negative as well.

**Figure 8:**
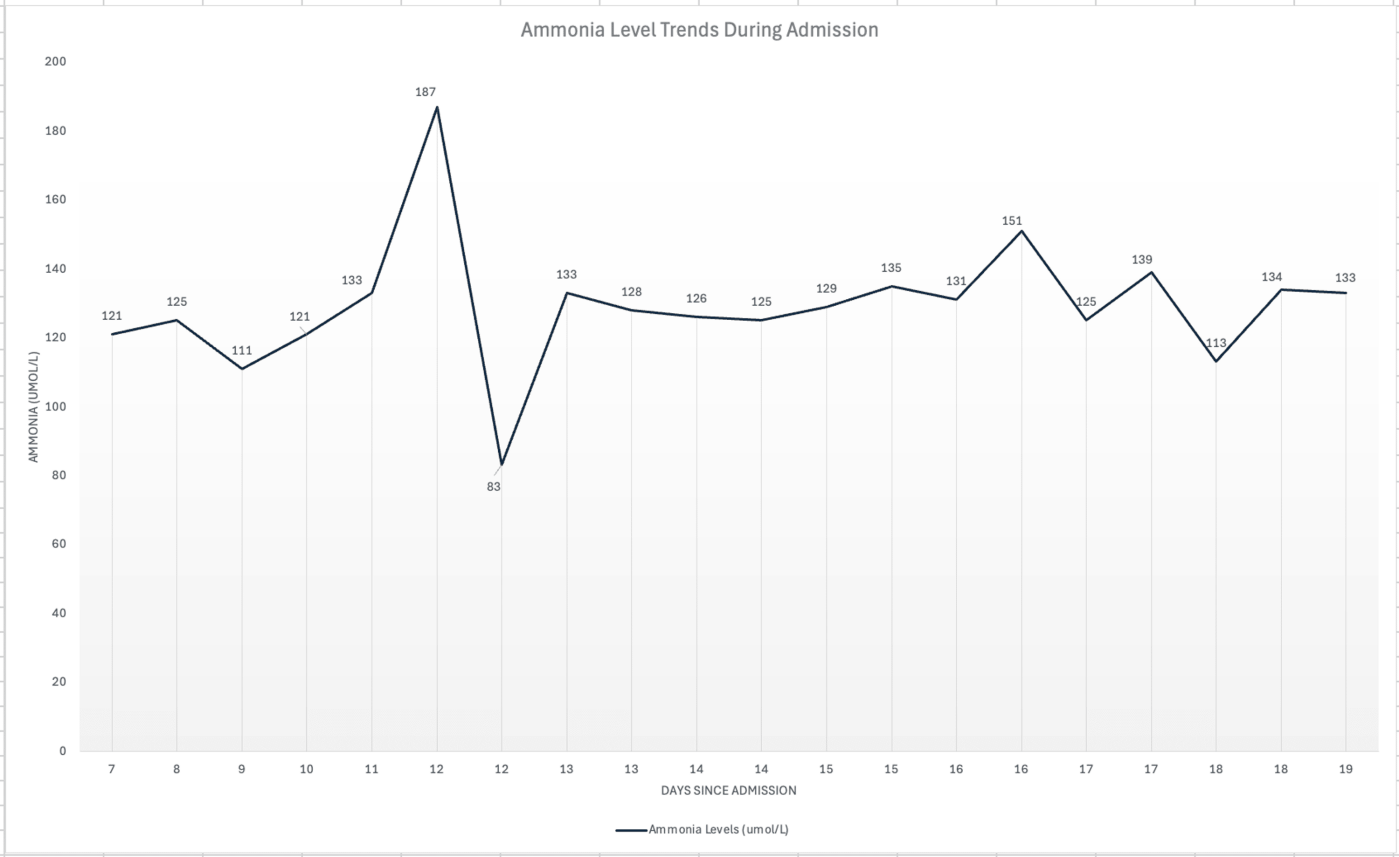
Ammonia level trends during admission Blue: ammonia levels in micromoles per liter (umol/L). Ammonia normal reference range = 18-72 umol/L.

**Figure 9:**
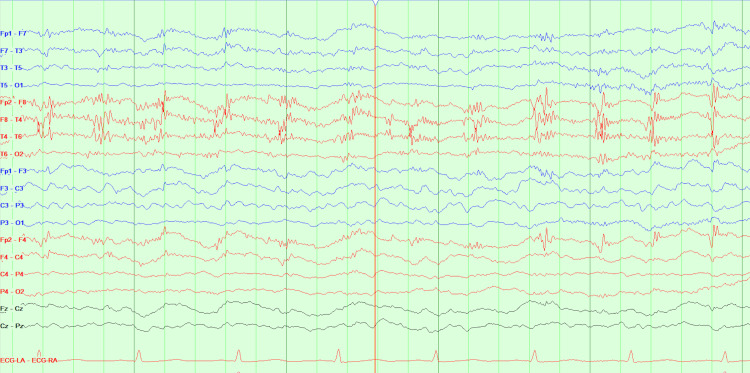
EEG demonstrating frequent multifocal sharp waves and focal electrographic seizures EEG = electroencephalogram

As a result of worsening mental status, ammonia levels were obtained, which were elevated at 121 umol/L (Figure 8, day 7). The patient was subsequently treated with lactulose and rifaximin, which did not improve ammonia levels, as repeat ammonia was 125 umol/L (Figure 8, day 8). Despite maintenance fluids, the patient’s sodium levels increased significantly from 141 mmol/L to 156 mmol/L overnight in the additional context of having repeated episodes of diarrhea while continuing to have no improvement in mental status. Arterial blood gas at this time was significant for a pH of 7.53, PaCO_2_ of 21 mmol/L, and bicarbonate of 20.2 mmol/L with a normal respiratory rate. In the setting of worsening mental status and a Glasgow Coma Scale of 9, the patient was then transferred to the medical intensive care unit for further management and was intubated due to the inability to protect their airway. The patient was trialed on continuous venovenous hemodiafiltration that only resulted in a minimal reduction in ammonia from 128 umol/L to 125 umol/L (Figure 8, days 13-14). She was then switched to intermittent hemodialysis with an unsuccessful attempt to reduce bicarbonate. With continued altered mental status and lack of responsiveness, a repeat EEG (Figure 9) revealed frequent multifocal sharp waves and focal electrographic seizures, which were treated with levetiracetam at 1 gram twice daily. Unfortunately, the patient remained unresponsive after extubation, and following consultations with palliative care, the family decided to transition to hospice care and comfort measures.

## Discussion

The most common causes of encephalopathy in MM are hypercalcemia, hyper-viscosity, uremia, and direct osseous leptomeningeal involvement [6]. Hyperammonemia is a cause of altered mental status in MM and occurs in less than 1%, making diagnostic evaluation difficult to approach early on [7]. It is important to rule out other common causes of hyperammonemia, like liver failure, kidney failure, parenteral nutrition, urinary tract infection with urease-producing organisms, and medication overdoses like valproic acid, which can happen in MM patients [8]. Given the rarity of hyperammonemia in MM patients, its underlying mechanism remains poorly understood. What is evident, however, is that this uncommon presentation is often associated with poor patient outcomes [5]. One pathophysiologic theory of hyperammonemia in MM is that some specific subtypes of multiple myeloma may experience leukemic changes that increase the likelihood of ammonia development [5]. Of the various subtypes, IgG and IgA MM are seen the most frequently at 40% and 35% of cases demonstrating hyperammonemia [5]. On the other hand, there are reports of plasmablastic myeloma demonstrating hyperammonemic encephalopathy as well [6]. Some studies have shown that in vitro ammonia production was significantly increased in myeloma cell lines than in non-myeloma cells. The proposed mechanism is an increase in the protein synthesis of immunoglobulins and cytokines, increasing ammonia production [9]. Another potential mechanism proposed is that plasma cell infiltration into hepatic tissues results in porto-systemic shunting of blood, leading to an increase in ammonia production [5]. Hyperammonemia can even be seen after high doses of chemotherapy in MM patients [10]. With a limited understanding of how ammonia is produced in MM, it becomes challenging to identify effective strategies to target its accumulation.

In one of the studies, it was observed that six out of 85 MM patients had elevated ammonia levels with no known etiology of hyperammonemia and compared the abnormal lab findings of these patients with patients of hyperammonemia with liver disease. It was reported that although the level of hyperammonemia was similar in both MM and chronic liver disease patients, the level of individual amino acids was different, the level of glycine being higher in MM patients and the level of tyrosine being higher in chronic liver disease patients. Moreover, a substantial decrease in branched-chain amino acids like leucine, isoleucine, and valine was noted in MM cases [11].

In patients with hyperammonemia who receive therapy for MM, the mortality rate is as high as 48% while those without therapy have all died according to current literature [5]. Therapeutic interventions reported in the literature include proteasome inhibitors, steroids, and immunomodulators [1]. Our patient was also on this regimen with carfilzomib, pomalidomide, and dexamethasone. Overall, chemotherapy tends to result in improved outcomes [5]. However, there are additional reports of hyperammonemic encephalopathy after receiving high-dose chemotherapy such as daratumumab [10]. One of the cases reported improvement of mental status and normalization of ammonia in a patient receiving daratumumab, bortezomib, and dexamethasone [5]. Some success has been achieved in improving mental status with melphalan [5].

A common approach to improving ammonia-induced encephalopathy is to give lactulose; however, lactulose has been seen to provide no improvement in some patients [5]. This could potentially be explained by the fact that the source of hyperammonemia in MM is not from the gut, but instead from other mechanisms, and lactulose only works on the gut by intraluminally countering ammonia production by bacteria. Hemodialysis represents another avenue to remove ammonia from the body. Of the small population of reported cases, supportive hemodialysis did improve mental status in 80% of patients, but these patients additionally had concurrent therapy for multiple myeloma [5]. Other literature, however, has shown that despite the improvement in mental status, ammonia levels quickly return to high levels between dialysis treatments [10]. This lack of a standardized, approved management plan makes treatment highly difficult, along with the goal of achieving sustained improvement for patients with this rare complication.

Another potential cause of metabolic alteration in MM may stem from its unique dependence on glutamine. MM plasma cells are particularly reliant on extracellular glutamine for their proliferation and survival. Notably, many MM cells lack expression of glutamine synthetase, the enzyme that synthesizes glutamine from glutamate and ammonia, yet exhibit high levels of glutaminase, which breaks down glutamine [12]. As a result, MM patients often display low intracellular levels of glutamine alongside elevated glutamate and ammonia concentrations [12,13].

Glutamine has traditionally been considered a non-toxic carrier of ammonia in the body; however, emerging hypotheses suggest that glutamine can be transported into mitochondria, where it is hydrolyzed to release ammonia, potentially causing mitochondrial dysfunction and oxidative damage [13]. Furthermore, the imbalance of low glutamine and high glutamate levels has been shown to induce glutamine synthetase expression in mesenchymal stromal cells. This induction impairs osteoblast differentiation, contributing to the characteristic bone lesions observed in MM patients [12]. Thus, MM plasma cells are not only heavily dependent on extracellular glutamine, but their metabolism also contributes to increased ammonia production, further driving pathological features of the disease. This has led to therapeutic considerations of how to decrease glutamine production in order to counteract these MM cells that are highly dependent on glutamine. Certain studies have shown that glutaminolysis and deprivation of glutamine in the culture medium of plasma and leukemia cells lead to cell cycle arrest and inhibition of cellular growth of these cell types. These same studies also revealed that the inhibition of glutamine synthetase can detrimentally impact the survival impact myeloma cell lines and can increase the cytotoxic effects of HDAC inhibitors, which are commonly used in multiple myeloma treatment [14]. However, a significant challenge remains: both glutaminolysis and inhibition of glutamine synthetase may further increase ammonia accumulation. This highlights the complexity of targeting glutamine metabolism in MM and underscores the difficulty of managing hyperammonemia in these patients.

## Conclusions

Hyperammonemia in the context of MM is a rare but life-threatening complication that requires early recognition and prompt intervention. The rapid progression of encephalopathy due to elevated ammonia levels can quickly lead to coma and death, underscoring the urgency of diagnosis and treatment. While therapeutic strategies such as chemotherapy to reduce myeloma burden, ammonia-lowering agents, and supportive measures like hemodialysis have been attempted, their efficacy remains inconsistent. As noted previously, existing literature suggests that survival is markedly better among patients who receive myeloma-directed therapy as compared to those who only receive supportive care. However, literature also suggests that myeloma-directed therapy may also contribute to encephalopathy. 

The variety of approaches reported in the literature and the lack of randomized controlled trials make establishing a definitive management and treatment plan for hyperammonemia in MM particularly challenging. Without standardized guidelines, clinicians must rely on case reports and individualized treatment strategies, often leading to variability in patient outcomes. This highlights the need for further investigation into the pathophysiology of MM-associated hyperammonemia to develop more targeted and effective treatment approaches.

Given the poor prognosis associated with this condition, better prognostic stratification by intervention type and increased awareness amongst clinicians is essential. Identifying at-risk patients and recognizing early neurological symptoms may allow for timely interventions that could potentially improve outcomes. Future research should focus on elucidating the underlying mechanisms of hyperammonemia in MM and developing standardized, evidence-based treatment protocols to optimize patient care.
